# Surgical Outcomes of Ahmed or Baerveldt Tube Shunt Implantation for medically Uncontrolled Traumatic Glaucoma

**DOI:** 10.5005/jp-journals-10008-1215

**Published:** 2017-01-18

**Authors:** Arkadiy Yadgarov, Dan Liu, Elliot S Crane, Albert S Khouri

**Affiliations:** 1 Resident, Institute of Ophthalmology and Visual Science, New Jersey Medical School, Rutgers University, Newark, New Jersey USA; 2 Student, Institute of Ophthalmology and Visual Science, New Jersey Medical School, Rutgers University, Newark, New Jersey USA; 3 Student, Institute of Ophthalmology and Visual Science, New Jersey Medical School, Rutgers University, Newark, New Jersey USA; 4 Associate Professor, Institute of Ophthalmology and Visual Science, New Jersey Medical School, Rutgers University, Newark, New Jersey USA

**Keywords:** Cohort study, Glaucoma, Glaucoma drainage device, Intraocular pressure, Trauma, Tube.

## Abstract

**Aim:**

To describe postoperative surgical success of either Ahmed or Baerveldt tube shunt implantation for eyes with medically uncontrolled traumatic glaucoma.

**Materials and methods:**

A review was carried out to identify patients with traumatic glaucoma that required tube shunt implantation between 2009 and 2015 at Rutgers University in Newark, New Jersey, USA. Seventeen eyes from 17 patients met inclusion criteria, including at least 3-month postoperative follow-up. The main outcome measure was surgical success at 1-year follow-up after tube implantation.

**Results:**

Mean preoperative intraocular pressure (IOP) was 34.1 ± 8.2 mm Hg on 3.1 ± 1.6 ocular hypotensive medications. Nine eyes (53%) sustained closed globe injury. Ten eyes (59%) received an Ahmed valve shunt and seven eyes (41%) received a Baerveldt tube shunt. Surgical success rate at 1 year postoperatively was 83%. Compared to preoperative, the mean postoperative IOP was significantly lower (16.1 ± 3.5 mm Hg, p < 0.001) on significantly fewer ocular hypertensive medications (1.3 ± 1.6, p = 0.001) at a mean follow-up of 10 months. Mean IOP reduction at last follow-up was 49%. There were three cases of surgical failures: One case of hypotony, one case of tube extrusion with subsequent explan-tation, and one case requiring second tube insertion for IOP control.

**Conclusion:**

Implantation of an Ahmed or Baerveldt tube shunt provided successful control of IOP in patients with medically uncontrollable traumatic glaucoma.

**How to cite this article:**

Yadgarov A, Liu D, Crane ES, Khouri AS. Surgical Outcomes of Ahmed or Baerveldt Tube Shunt Implantation for medically Uncontrolled Traumatic Glaucoma. J Curr Glaucoma Pract 2017;11(1):16-21.

## INTRODUCTION

Globe trauma is a known cause of secondary glaucoma. Elevated intraocular pressure (IOP) can develop after globe injury through different possible mechanisms, which include hyphema, synechial angle closure, lens injury, direct trabecular meshwork injuries with or without angle recession, and inflammation.^1^ The overall incidence of traumatic glaucoma has been reported to be 2 to 17%, depending on follow-up and diagnostic criteria.^2-5^ Management is aimed at preventing further damage to the injured eye. Early-onset traumatic glaucoma is commonly secondary to inflammation, hyphema, and/or lens injury and generally resolves with medicine, such as ocular hypotensives, corticosteroids, anterior chamber washout, and/or lens removal.^5-7^ Late-onset traumatic glaucoma is mostly associated with angle recession glaucoma or closed angle glaucoma and more prone to necessitate incisional glaucoma surgery.^1,7^ Trabeculectomy with mitomycin C or tube shunt implantation are the most commonly utilized surgical options. Angle recession and trauma are reported risk factors for trabeculectomy failure.^8,9^ Additionally, eyes with the history of globe injury may be more likely to be pseudophakic or aphakic, both independent risk factors for trabeculectomy failure.^10,11^ Although addition of mitomycin C to trabeculectomy has been shown to provide adequate IOP control and better postoperative success rate with angle recession glaucoma, exposure to mitomycin C is associated with an increased risk of infection.^8,12^ Since traumatic glaucoma patients are younger and have a long life expectancy, the risk of late bleb infection is relatively higher.^13^ Tube shunt surgery is an appropriate option due to acceptable IOP success rates without an increased risk for infection.^14,15^ While successful control of IOP in traumatic glaucoma has been clearly demonstrated with Molteno tube shunt implantation, surprisingly there are very few studies that report surgical outcomes after Ahmed or Baerveldt tube implantation for traumatic glaucoma, with the largest series consisting of six cases.^5,15^ Our case series is the largest to date to report on surgical outcomes of eyes with traumatic glaucoma that underwent either Ahmed or Baerveldt tube shunt implantation.

## MATERIALS AND METHODS

This study was approved by the Institutional Review Board at Rutgers University Hospital, Newark, New Jersey, USA, and was ethically conducted in accordance with the Declaration of Helsinki. A retrospective chart review was conducted on patients who were diagnosed with traumatic glaucoma and underwent tube shunt implantation during the period from January 2009 to January 2015 at Rutgers University Hospital. Inclusive criteria were patients with a confirmed history of trauma, persistent elevated IOP of >21 mm Hg for ≥2 consecutive visits after trauma despite maximal medical therapy, underwent either Ahmed or Baerveldt tube shunt surgery, and had a minimum follow-up of 3 months postoperatively. Patients with a history of glaucoma or glaucoma suspicion before or unrelated to trauma were excluded. Eyes with a history of globe injury were differentiated into two categories, open globe injury (OGI) and closed globe injury (CGI). An OGI was defined as a traumatic full-thickness wound in the cornea and/or sclera, whereas a CGI was defined as an ocular contusion. Open globe injury eyes were further classified by zones of injury based on the open globe classification system described by Pieramici et al.^16^ Briefly, the zone of injury is determined by the location of the most posterior full-thickness wound, which is isolated to the cornea in Zone I, the anterior 5 mm of the sclera in Zone II, and the remaining posterior sclera in Zone III.^16^ Eyes that presented with an OGI were surgically repaired within 24 hours at our institution. A data collection sheet that included demographic data and clinically relevant information was created. Data recorded included patient age, gender, mechanism of injury, zone of injury (for OGI eyes), associated ocular damage, and medical and surgical interventions following trauma including tube shunt implantation. The preoperative IOP for each eye was recorded as the mean IOP in the immediate two visits before tube shunt surgery. Preoperative visual acuity and preoperative medications were recorded from the visit immediately before tube shunt surgery. After surgery, IOP and glaucoma medications were recorded as the mean for visits within timeframes: <1 month, 1 to 3 months, 6 to 11 months, and 1 year postopera-tively. The primary outcome measure was success rate at 1 year postoperatively. Success was defined as achieving postoperative IOP of ≤21 mm Hg with or without ocular hypotensive medication. Failure was defined as an eye with IOP (>21 mm Hg) on at least two consecutive visits, while on maximum tolerated glaucoma medications, persistent hypotony (IOP < 6 mm Hg at ≥2 consecutive visits), tube explantation, progression to no light perception (NLP) vision, or eyes that underwent diode cyclophotocoagulation (CPC). Tube revision was not considered a failure in this study.

Unless otherwise specified, statistical analysis was carried out using a paired two-tailed t-test to compare changes between preoperative and postoperative IOP and number of ocular medications. Means and standard deviations (SD) of visual acuity were calculated after first converting all Snellen values to logMAR; visual acuities of count fingers, hand motion, light perception, and NLP were considered logMAR 1.6, 2.0, 2.5, and 3.0 respectively.

### Surgical Procedure

Tube surgery was performed by one of three surgical glaucoma specialists. Surgeon preference dictated the use of either the Ahmed FP7 Glaucoma Valve or the Baerveldt 101-350 Glaucoma Implant. For Baerveldt tube implants, all surgeons used a 4-0 polypropylene suture as an intraluminal stent, which was externalized, buried in the inferior fornix and removed at a later date. Based on surgeon preference, some Ahmed and Baerveldt tubes were ligated with a polyglactin suture. No antime-tabolites were used intraoperatively or postoperatively.

## RESULTS

Over a 6-year study period of January 2009 to January 2015, 40 eyes with traumatic glaucoma that underwent tube shunt implantation were identified. Among those subjects, 17 patients (all unilaterally affected) met study inclusion criteria. Of the 17 patients, 16 patients (94%) were male. All patients with a history of OGI were male. The mean age was 43.6 ± 18.3 with age range between 13 and 78 years. More left eyes were affected (12 eyes, 71%) than right eyes. Nine eyes had a history of CGI while eight had a history of OGI. The most common mechanism of injury was a projectile object (12 eyes, 71%) (Table 1). Six cases (75%) of OGI were due to penetrating globe injury, while only two cases were due to globe rupture. Among eyes with OGI, five eyes had a zone 1 injury and three eyes had a zone two injury. The most commonly associated ocular injuries included lens injury (seven cases), hyphema (six cases), vitreous hemorrhage (six cases), and retinal detachment (three cases). There was one case of intraocular foreign body. Average ocular surgeries performed between globe injury and glaucoma intervention was 1.7 surgeries. Eyes with OGI had an expected higher average number of ocular surgeries (2.9 surgeries) compared to CGI (0.7 surgeries).

**Table 1:** **Table 1:** Cause of injury in eyes with traumatic glaucoma

*Type of injury*		*Total (n = 17)*		*CGI (n = 9)*		*OGI (n = 8)*	
Projectile		12		6		6	
Assault		2		2		0	
Accidental blunt injury		3		1		2	

**Graph 1:**
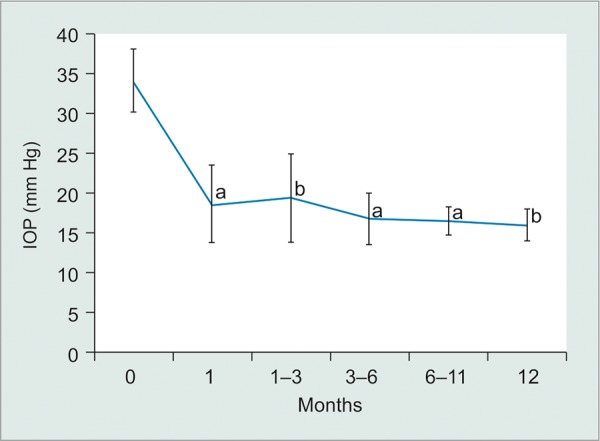
Average IOP values at preoperative and postoperative time points after tube shunt implantation (95% confidence error bars); p values compare preoperative and postoperative values. a: p < 0.0001; b: p < 0.001

**Graph 2:**
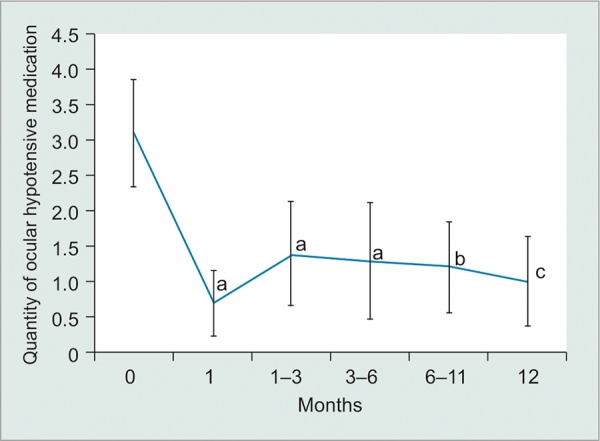
Average quantity of ocular hypotensive medications at preoperative and postoperative time points (95% confidence error bars); p values compare preoperative and postoperative values. a: p < 0.0001; b: p < 0.001; c: p = 0.001

At the examination prior to glaucoma surgical intervention, 59% of eyes were either aphakic or pseudophakic and visual acuity was worse than 20/200 in 82% of eyes at last preoperative visit. The median time from globe injury to glaucoma surgery was 19 months. The mean preoperative IOP was 34.1 ± 8.2 mm Hg on an average 3.1 ± 1.6 ocular hypotensive medications. The mean preoperative best-corrected visual acuity was 1.47 ± 0.47 logMAR (20/585 Snellen equivalent, or one line better than count fingers). Ahmed tubes were most commonly used (10/17, 59%). At a mean follow-up of 10 months, the average postoperative IOP was 16.1 ± 3.5 mm Hg on 1.3 ± 1.6 ocular hypotensive medications, and the mean postoperative visual acuity was 1.56 ± 0.71 logMAR (20/725 Snellen equivalent, or half a line better than count fingers). Table 2 summarizes these results along with information about subset groups based on type of globe injury and type of tube implant. There was a statistically significant lowering of both preoperative IOP and preoperative ocular hypotensive medicines at all postoperative time points (p < 0.001 and p < 0.001 respectively; Graphs 1 and 2). Mean IOP reduction at last follow-up postoperatively was 49%. Success was achieved in 82% of cases at mean follow-up of 10 months and 83% at 1 year postoperatively. There were four cases of tube revision. Reasons for revision included tube occlusion by iris, excessively long tube in the anterior chamber, tube exposure, and malpositioned wound. Three cases met failure criteria, one due to chronic hypotony, one case of tube extrusion requiring explantation and subsequent CPC, and one due to chronic hypertony requiring implantation of a second tube.

**Table 2:** **Table 2:** Preoperative characteristics and postoperative outcomes

		*Total (n = 17)*		*CGI (n = 9)*		*OGI (n = 8)*		*Ahmed (n = 10)*		*Baerveldt (n = 7)*	
Age (years)											
Mean ± SD		43.6 ± 18.3		41.9 ± 22.3		45.4 ± 13.7		52.4 ± 14.7		31.0 ± 16	
Sex											
M (%)		16 (94%)		8		8		9		7	
F		1		1		0		1		0	
Lens status^a^											
Phakic		7		5		2		5		2	
Pseudo- or aphakic (%)		10 (59%)		4		6		5		5	
Preoperative IOP (mm Hg)											
Mean ± SD		34.1 ± 8.2		37.5 ± 8.4		30.3 ± 6.7		32.2 ± 6.0		36.9 ± 10.6	
Preoperative Meds											
Mean ± SD		3.1 ± 1.6		2.6 ± 1.9		3.7 ± 0.9		2.9 ± 1.7		3.4 ± 1.4	
Postoperative IOP^b^ (mm Hg)											
Mean ± SD		16.1 ± 3.5		17.3 ± 3.2		14.6 ± 3.6		15.9 ± 3.5		16.3 ± 3.8	
Postoperative Meds^b^											
Mean ± SD		1.3 ± 1.6		1.1 ± 1.7		1.0 ± 0.8		1.8 ± 1.8		0.7 ± 1.2	
Tube revisions (%)		4 (20%)		4		0		2		2	
Surgical failure (%)		2 (12%)		1		1		2		0	

^a^At the time of glaucoma surgery; ^b^At last follow-up (mean = 10 months); Meds: Medications

## DISCUSSION

To our knowledge, this study is the largest to report outcomes of Ahmed or Baerveldt tube shunt implantation in eyes with medically uncontrolled traumatic glaucoma. Traumatic glaucoma encompasses a spectrum of disease that depends on degree and extent of injured ocular tissues. Early-onset glaucoma is due to inflammation, trabecular meshwork disruption, and hyphema and generally resolves with medical treatment.^1^ Both Turalba et al^5^ and Ozer et al^6^ described successful control of approximately 75% of cases using only medical treatment for early-onset traumatic glaucoma. Additionally, early-onset glaucoma caused by retained lens particles, a cataract, or hyphema may require surgical intervention with lens particle removal, cataract extraction, and/or anterior chamber washout respectively. In a study by Osman et al,^17^ 48% of 31 eyes that needed surgical intervention were successfully controlled with either lens aspiration (with or without vitrectomy) or anterior chamber washout. In contrast, traumatic glaucoma that develops greater than 6 months after globe injury (delayed-onset glaucoma) is less common and is less amenable to medical interven-tion.^1,7^ Delayed-onset glaucoma is typically described as a chronic angle recession glaucoma, especially after CGI, or chronic closed angle glaucoma secondary to peripheral anterior synechiae, especially after OGI.^1^ Surgical management with trabeculectomy or tube shunt implantation is required to prevent further vision loss from medically uncontrolled glaucoma.^6,7,17^

Traumatic glaucoma typically affects a younger population compared to primary adult-onset glaucoma. In multiple studies of traumatic glaucoma, the average age ranged from 35 to 45 years.^2,3,8,12,14,15^ Similarly, the average age of patients in our study at the time of incisional glaucoma surgery was 43.6 years. Since trabeculectomy with mitomycin C confers a greater risk of late-onset infection in patients with a longer expectation of life, tube shunt implantation may be a safer alternative.^8,13^

In our study, 17 eyes received either an Ahmed or Baerveldt tube shunt. More than 90% of patients in the study were male, a demographic consistent with many previously reported studies.^2,3,8,15^ Ten eyes (59%) were either aphakic or pseudophakic at last preoperative visit. Fourteen eyes (82%) had a preoperative visual acuity worse than 20/200 secondary to various causes, including corneal pathology, aphakia, vitreous debris, macular pathology, and/or optic nerve pathology. Our study did not focus on visual outcomes after tube shunt implantation due to various confounding ocular comorbidities commonly associated with globe injury. As demonstrated by Turalba et al,^5^ visual outcome after globe injury is more related to factors other than elevated IOP. In that study, there was an improvement in vision from a median preoperative visual acuity of hand motions to a median postoperative visual acuity of 20/60. The authors noted that visual acuity likely improved from the treatment and resolution of comorbid eye conditions, such as traumatic cataract, corneal laceration, and hyphema. The aim of traumatic glaucoma management is to prevent further nerve damage, thus reduction of IOP to physiologic levels was emphasized in our study, not visual outcome. No eyes in our study progressed to NLP vision, a criteria for surgical failure.

Various studies have attempted to identify risk factors associated with development of traumatic glaucoma. Although risk factors vary between studies and differ based on type of globe injury, the most common risk factors reported were older age, presenting visual acuity worse than 20/200, intraocular bleeding (vitreous hemorrhage or hyphema), and lens injury.^2-7^ In our study, lens injury and intraocular hemorrhage were the most common ocular comorbidities. Additionally, as mentioned above, 82% of eyes had visual acuities worse than 20/200.

The mean preoperative IOP was 34.1 ± 8.2 mm Hg in our case series, which compares well to a study by Fuller et al^15^ that reported a preoperative IOP of 34.6 ± 12.4 mm Hg in 38 eyes. In our study, eyes with CGI demonstrated a higher mean preoperative IOP compared to eyes with OGI (37.5 mm Hg *vs* 30.3 mm Hg), although this did not reach statistical significance (p = 0.069, two-tailed, unpaired t-test).

The mean postoperative IOP was 16.1 ± 3.5 mm Hg at a mean follow-up of 10 months. Fuller et al^15^ described a mean postoperative IOP of 16.8 ± 3.6 mm Hg at 1 year with Molteno tubes. In our series, the mean postoperative IOP was similar in eyes with either OGI or CGI history. Fuller et al^15^ had equal representation of eyes with OGI and CGI and also reported no difference in postoperative success rate.

The success rate of tube shunt surgery for traumatic glaucoma was 82% at mean follow-up of 10 months. The 1-year postoperative success rate was 83% for eyes that completed 1-year of follow-up (n = 12). Three cases were surgical failures due to either chronic hypotony, hyper-tony, or tube extrusion with subsequent tube explantation.

A literature review on surgical outcomes of tube shunt implantation for traumatic glaucoma yields studies primarily using Molteno tubes. Mermoud et al^14^ showed that a single-plate Molteno tube was able to successfully control IOP in 46% of 12 traumatic angle recession cases at a mean follow-up of 16 months. Mills et al^18^ reported a long-term overall success rate of 63% in eight eyes with a median follow-up of 51 months also using Molteno single-plate implants. A prospective study by Fuller et al^15^ of 38 eyes that underwent Molteno implantation for traumatic glaucoma reported an overall success rate of 76% at a mean follow-up of 10.9 years. About 85% of the tubes used in that study were double plate Molteno implants. Fuller et al^15^ reported a 30% tube revision rate. Similarly our case series demonstrated a 24% tube revision rate. There were no implant-related infections reported in the studies with Molteno tubes and likewise none in our case series.

**Graph 3:**
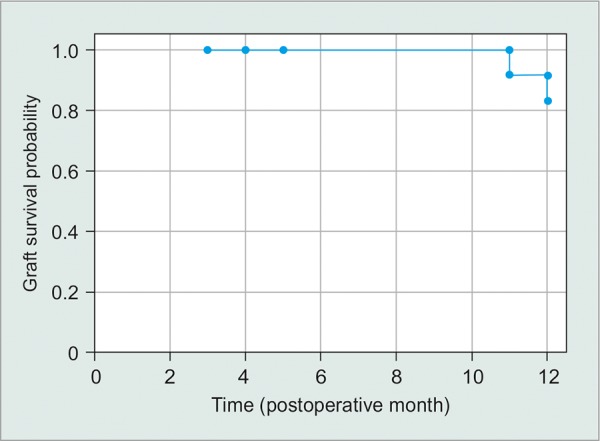
Kaplan - Meier survival curve for graft survival

In the past decade, both Baerveldt and Ahmed tube shunts have become widely used shunts for various ocular conditions.^19^ There have been only a few case series to report outcome on either Baerveldt or Ahmed tubes for traumatic glaucoma. Osman et al^17^ implanted two Ahmed shunts for traumatic glaucoma and reported a 100% success rate over a 12-month mean follow-up. Turalba et al^5^ also reported a 100% surgical success rate in six cases (five Ahmed tubes and one Baerveldt tube) at 1-year follow-up. Our case series of 17 eyes is the largest to report on outcomes of traumatic glaucoma after either Ahmed or Baerveldt tube implantation. We demonstrate an 82% success rate at a mean follow-up of 10 months in 17 eyes. At 1 year, the success rate was 83% in 12 eyes that were present for 1-year follow-up. The success rate at all time points postoperatively remained relatively high and stable (Graph 3).

Limitations of our study include its retrospective design, which introduces patient and selection biases. Another limitation was the small quantity of cases recruited. Thus, there was insufficient power to differentiate statistically significant differences between subsets of type of globe injury and type of tube shunt implant used. The incidence of traumatic glaucoma is low; two studies by Girkin et al^2,3^ reported a 6-month incidence rate of 2.67 to 3.39% in a combined 9,648 trauma cases. Since, as described above, a majority of traumatic glaucoma cases recover with medical treatment, it becomes difficult to recruit enough patients that fit criteria and have adequate follow-up. Fuller et al^15^ prospectively collected 38 cases over a 20-year period. Our study was limited to a 6-year retrospective review period because of a 6-year recall limit of diagnostic and procedural codes from the billing department database.

The latency for needing glaucoma surgery may be very long in some patients. While the mean time between globe injury and glaucoma surgery was 19 months in our study, Fuller et al^15^ reported a median time of 10 years between globe injury and tube shunt surgery; likewise, Mermoud et al^8^ reported a mean period of 7 years. This fact emphasizes the importance of long-term monitoring of eyes with globe trauma.

Management of traumatic glaucoma is important to prevent further vision loss and surgical intervention is necessary when medical therapy fails. Our study demonstrates a high surgical success rate at 1 year using either Ahmed or Baerveldt tube shunts.

## CONCLUSION

Implantation of an Ahmed or Baerveldt tube shunt provided successful control of IOP in patients with medically uncontrollable traumatic glaucoma.

## CLINICAL SIGNIFICANCE

Successful IOP control in traumatic glaucoma has been demonstrated following Molteno tube shunt implantation. This study similarly demonstrates that Ahmed and Baerveldt tube shunts can provide successful IOP control in cases of traumatic glaucoma.

## References

[1] De Leon-Ortega JE, Girkin CA (2002). Ocular trauma-related glaucoma.. Ophthalmol Clin North Am.

[2] Girkin CA, McGwin G Jr, Long C, Morris R, Kuhn F (2005). Glaucoma after ocular contusion: a cohort study of the United States Eye Injury Registry.. J Glaucoma.

[3] Girkin CA, McGwin G Jr, Morris R, Kuhn F (2005). Glaucoma following penetrating ocular trauma: a cohort study of the United States Eye Injury Registry.. Am J Ophthalmol.

[4] Osman EA, Al-Fawaz N, Al-Otaibi AG, Al-Mansouri SM, Mousa A, Al-Mezaine HS (2013). Glaucoma after open globe injury at a tertiary care university hospital in Central Saudi Arabia. Cumulative incidence and risk factors.. Saudi Med J.

[5] Turalba AV, Shah AS, Andreoli MT, Andreoli CM, Rhee DJ (2014). Predictors and outcomes of ocular hypertension after open-globe injury.. J Glaucoma.

[6] Ozer PA, Yalvac IS, Satana B, Eksioglu U, Duman S (2007). Incidence and risk factors in secondary glaucomas after blunt and penetrating ocular trauma.. J Glaucoma.

[7] Bai HQ, Yao L, Wang DB, Jin R, Wang YX (2009). Causes and treatments of traumatic secondary glaucoma.. Eur J Ophthalmol.

[8] Mermoud A, Salmon JF, Barron A, Straker C, Murray AD (1993). Surgical management of post-traumatic angle recession glaucoma.. Ophthalmology.

[9] Mermoud A, Salmon JF, Straker C, Murray AD (1993). Post-traumatic angle recession glaucoma: a risk factor for bleb failure after trabeculectomy.. Br J Ophthalmol.

[10] Takihara Y, Inatani M, Ogata-Iwao M, Kawai M, Inoue T, Iwao K, Tanihara H (2014). Trabeculectomy for open-angle glaucoma in phakic eyes vs in pseudophakic eyes after phaco-emulsification: a prospective clinical cohort study.. JAMA Ophthalmol.

[11] Landers J, Martin K, Sarkies N, Bourne R, Watson P (2012). A twenty-year follow-up study of trabeculectomy: risk factors and outcomes.. Ophthalmology.

[12] Manners T, Salmon JF, Barron A, Willies C, Murray AD (2001). Trabeculectomy with mitomycin C in the treatment of post-traumatic angle recession glaucoma.. Br J Ophthalmol.

[13] DeBry PW, Perkins TW, Heatley G, Kaufman P, Brumback LC (2002). Incidence of late-onset bleb-related complications following trabeculectomy with mitomycin.. Arch Ophthalmol.

[14] Mermoud A, Salmon JF, Straker C, Murray AD (1992). Use of the single-plate Molteno implant in refractory glaucoma.. Ophthalmologica.

[15] Fuller JR, Bevin TH, Molteno AC (2001). Long-term follow-up of traumatic glaucoma treated with Molteno implants.. Ophthalmology.

[16] Pieramici DJ, Sternberg P Jr, Aaberg TM Sr, Bridges WZ Jr, Capone A Jr, Cardillo JA, de Juan E Jr, Kuhn F, Meredith TA, Mieler WF (1997). A system for classifying mechanical injuries of the eye (globe). The Ocular Trauma Classification Group.. Am J Ophthalmol.

[17] Osman EA, Mousa A, Al-Mansouri SM, Al-Mezaine HS (2016). Glaucoma after open-globe injury at a Tertiary Care University Hospital: cumulative causes and management.. J Glaucoma.

[18] Mills RP, Reynolds A, Emond MJ, Barlow WE, Leen MM (1996). Long-term survival of Molteno glaucoma drainage devices.. Ophthalmology.

[19] Budenz DL, Barton K, Feuer WJ, Schiffman J, Costa VP, Godfrey DG (2011). Buys YM; Ahmed Baerveldt Comparison Study Group. Treatment outcomes in the Ahmed Baerveldt Comparison Study after 1 year of follow-up.. Ophthalmology.

